# Reorganising the emergency department to manage the COVID-19 outbreak

**DOI:** 10.1186/s12245-020-00294-w

**Published:** 2020-06-17

**Authors:** Li Juan Joy Quah, Boon Kiat Kenneth Tan, Tzay-Ping Fua, Choon Peng Jeremy Wee, Chin Siah Lim, Gayathri Nadarajan, Nur Diana Zakaria, Shi-En Joanna Chan, Paul Weng Wan, Lin Tess Teo, Ying Ying Chua, Evelyn Wong, Anantharaman Venkataraman

**Affiliations:** 1 Department of Emergency Medicine, Singapore General Hospital, 1 Outram Road, Singapore, 169608 Singapore; 2 Department of Infectious Diseases, Singapore General Hospital, 1 Outram Road, Singapore, 169608 Singapore

**Keywords:** COVID-19, Emergency department

## Abstract

**Background:**

The COVID-19 disease outbreak that first surfaced in Wuhan, China, in December 2019, has taken the world by storm and ravaged almost every country in the world. Emergency departments (ED) in hospitals are on the frontlines, serving an essential function in identifying these patients, isolating them early whilst providing urgent medical care. This outbreak has reinforced the role of Emergency Medicine in public health. This paper documents the challenges faced and measures taken by a tertiary hospital’s ED in Singapore, in response to the outbreak.

**Main body:**

The ED detected the first case of COVID-19 in Singapore on 22 January 2020 in a Chinese tourist and also the first case of locally transmitted COVID-19 on 3 February 2020. The patient journeys through the patient reception area in the ED and undergoes fever screening before being shunted to isolation areas within the ED. Management and disposition of suspect COVID-19 patients are guided by a close-knit collaboration between ED and department of infectious diseases. With increasing number of patients, back-up plans for expansion of space and staff augmentation have been enacted. Staff safety is also of utmost importance, with provision and guidelines for personal protective equipment and team segregation to ensure no cross-contamination across staff. These have been made possible with an early setup of an operational command and control structure within the ED, managing manpower, logistics, operations, communication and information management and liaison with other clinical departments.

**Conclusion:**

With the large numbers of undifferentiated patients managed by the ED to date, more than 820 patients with COVID-19 have been identified in the hospital. Not a single member of the staff of the SGH Emergency Department has come down with the illness. The various measures undertaken by the department have helped to ensure good staff morale and strict adherence to safety procedures. We share the lessons learnt so that others who manage EDs around the world can benefit from our experience.

## Background

The COVID-19 disease outbreak that first surfaced in Wuhan, China, in December 2019, has taken the world by storm and ravaged almost every country in the world [1]. Emergency departments (EDs), in most countries, have been on the frontline, meeting the undifferentiated patients presenting with a variety of complaints that represent the disease. Serving an essential function in identifying likely patients with the infection and isolating them early, this pandemic has reinforced the critical role played by Emergency Medicine in public health.

We document the measures taken by a busy ED in Singapore in managing the outbreak; outline the reasons for these various measures, their limitations, and details of implementation; and suggest approaches for the long-term role of Emergency Medicine in the community management of the COVID-19 pandemic and future communicable public health emergencies.

In reviewing these measures, we take a step-by-step approach using the patient’s pathway through the ED as a guide. We first describe the background against which the changes in the ED were implemented. Subsequently, we begin with the reception of patients and discuss screening procedures, triage, isolation, medical care in resuscitation, critical trolley areas, ambulatory patients, and the role of observation. Finally, we elaborate on the need for Emergency Medicine to take the lead in addressing screening of clusters of these infections and the role of surveillance research.

## The environment

Singapore is one of the smallest countries in the world. This tropical island country of 721.5 km^2^ had a population of 5.70 million people in 2019. Of these, 4.02 million are citizens and permanent residents. About 85% of the residents live in modern high-rise apartments scattered all over the island and the remaining 15% in landed properties [2]. Of the non-resident population, the labour force commands approximately 1.41 million persons, of whom around 323,000 are employed principally in the construction industry, living in crowded dormitories built by employers with financial assistance provided by the government [3].

Singapore’s healthcare system was ranked sixth in the World Health Organization’s (WHO) ranking of world health systems in 2000 and first by Bloomberg for the most efficient in the world in 2014 [4, 5]. Singapore has nine public hospitals with EDs. The 199-year-old Singapore General Hospital (SGH) is the oldest public healthcare institution in the country. It is also the largest, with 1785 beds in total. Its ED was established 72 years ago and manages approximately 130,000 patients annually. The ED has 70 medical staff (25 emergency physicians and 45 residents) and 200 registered nurses.

The first indication of the COVID-19 disease outbreak was in the city of Wuhan, Hubei Province, on 31 December 2019 when the Chinese government informed the WHO China country office about a cluster of 41 patients with a new respiratory infection. On 2 January, the Ministry of Health (MOH) in Singapore issued a health advisory and implemented temperature checks for passengers arriving at her international airport from Wuhan [6]. On 11 January 2020, the Chinese authorities informed the WHO that this infection was due to a novel coronavirus [7].

**Fig. 1:**
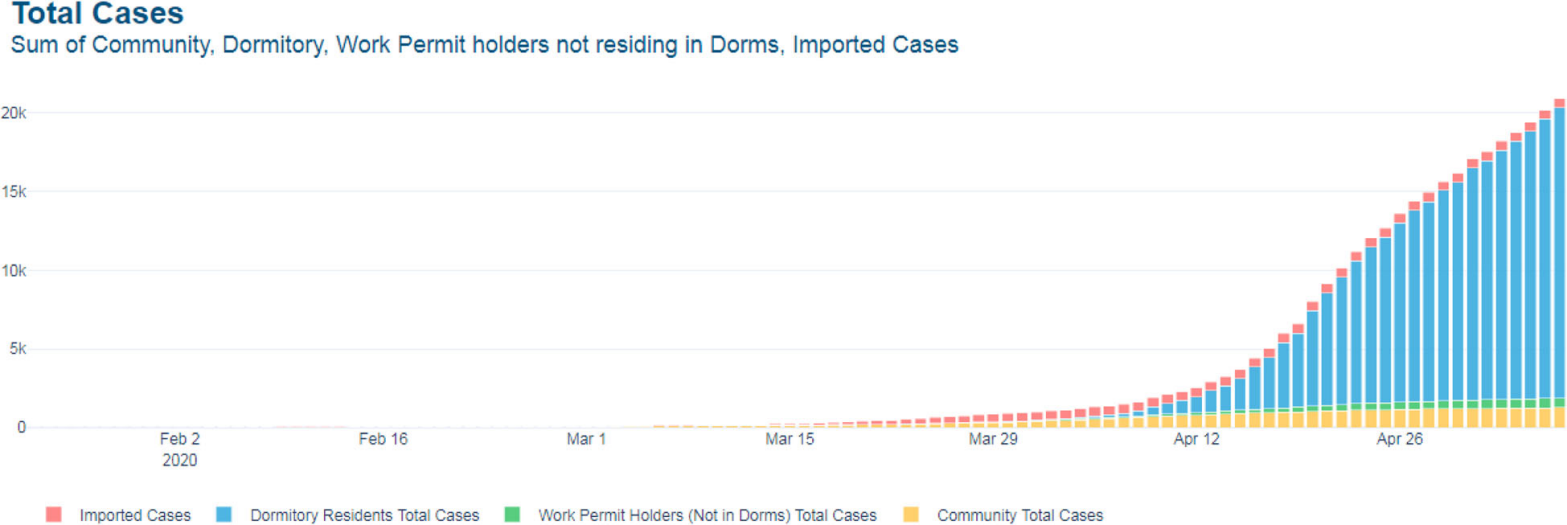
Graphic representation of dormitory workers contributing to national tally [9]

The first case of the illness in Singapore was in a tourist from Wuhan who presented to the ED at SGH on 22 January 2020. On 24 January 2020, the MOH declared the Disease Outbreak Response Condition (DORSCON) to be yellow. DORSCON is a colour-coded framework that shows the current infectious disease situation and provides guidelines on what can be done to prevent transmission and reduce the impact of infections. The colour codes vary from green (mild) to yellow (severe and minimal community spread), orange (severe and contained spread) and red (severe and spreading widely) [8]. The first instance of local transmission was picked up at the same ED on 3 February 2020. On 7 February 2020, the MOH upgraded the DORSCON situation to orange. The first COVID-19 death in Singapore was recorded on 24 March 2020. The first instances of foreign workers from the dormitories with the infection were on 30 March 2020 when 16 positive cases were identified. Just prior to that, Singapore had 844 COVID-19 cases, of whom all were either imported or from local transmission. Over the next few weeks, the number of COVID-19-positive cases amongst the foreign workers in the crowded dormitories surged to more than 700 a day and became a significant contributor to the national case tally (Fig. 1) On 7 April 2020, the government announced a state-wide lockdown (referred to as circuit breaker in Singapore) which has since been extended to 1 June 2020.

## Patient reception during COVID-19

**Fig. 2:**
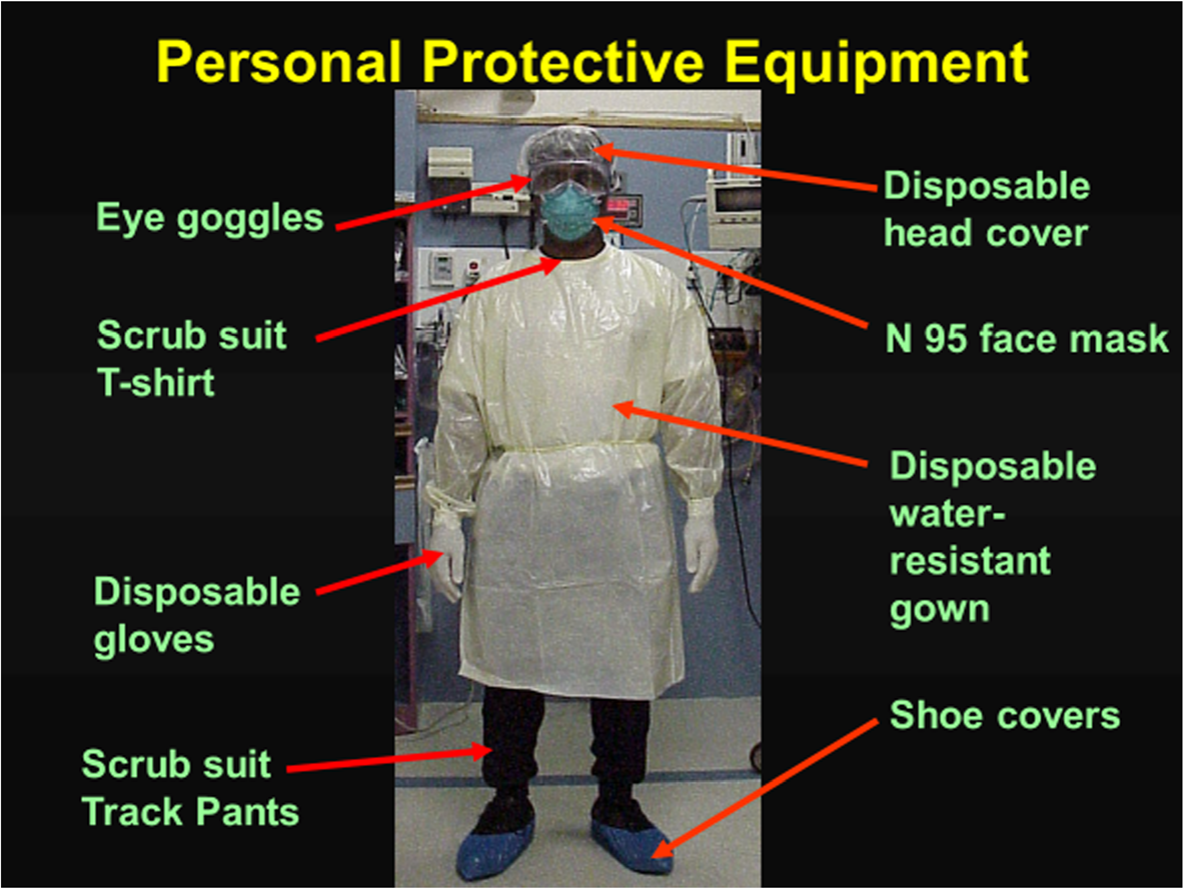
Staff in full personal protective equipment when attending to patients

Ever since the declaration of DORSCON orange, ambulance crew and hospital staff have donned personal protective equipment (PPE) with full-length gown, N95 masks and goggles when attending to patients (Fig. 2). All patients picked up are provided with surgical masks. When the patient is brought to the ED, reception staff transfer the patient to ED patient trolleys and conduct fever screening initially. The ambulance crew perform terminal cleaning of the interior of the ambulance and dispose of their gowns and gloves before leaving the ED for the ambulance stations. The fever screening process is described in the next section.

For patients arriving by transport means other than ambulances, the same group of fully gowned ED reception staff welcome and attend to them in a separate glass-enclosed fever screening facility at the entrance of the Department. From this location, the ED staff conduct the fever screening process. All these patients, as well as persons accompanying them, will also be provided with a surgical mask each. Only one person will be allowed to accompany the patient into the ED. Additional accompanying persons will be advised to wait in the cafeteria at the neighbouring hospital block.

The ED has two entrances, one at the front of the Department and one at the rear. At the onset of the DORSCON yellow period, the rear entrance was closed with a staff-card accessed door. This was to better ensure access control and that all patients and companions would only come in from the front of the Department.

## Fever screening

Fever screening was first introduced into the ED 17 years ago at the beginning of the SARS (severe acute respiratory syndrome) outbreak in March 2003. This was in response to the challenge of segregating high-risk patients from other patients early on in their patient journey and placing them in appropriate areas with airborne and droplet precautions addressed. This served to prevent spread to other patients and was also a reminder to attending healthcare staff that appropriate PPE should be donned before such patient contact. Since then, all patients coming to the ED undergo fever screening before they are triaged or formally registered at the Department.

**Fig. 3:**
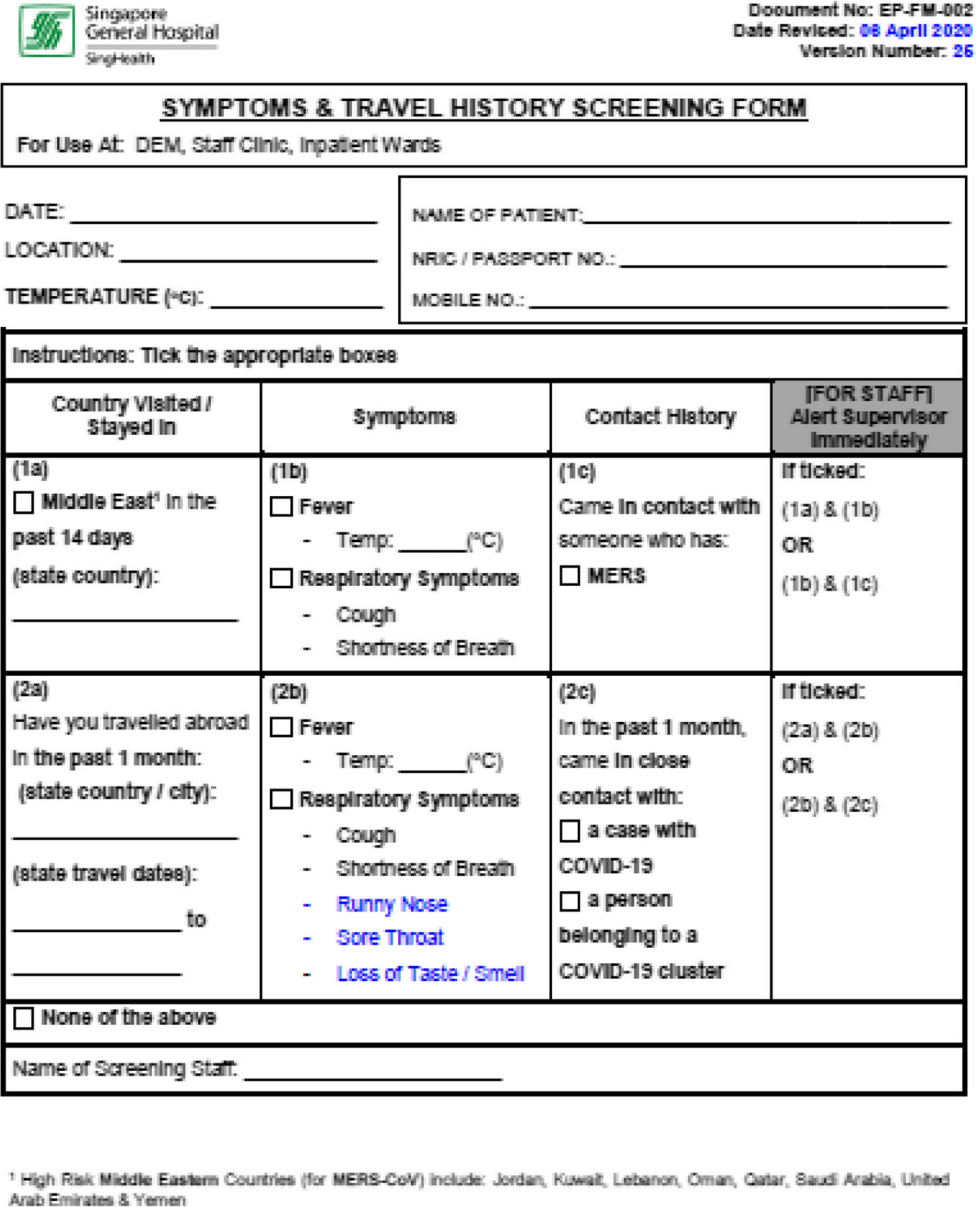
Fever screening form

During fever screening, a trained staff member will ask the patient to provide demographic and contact particulars, symptomology and travel and contact history. The contact particulars of the accompanying person(s) are also recorded. The temperature of the patient will be checked. These details will be documented in a fever screening form (Fig. 3). If the patient is deemed to have failed the screening (possibly likely to have a communicable infectious disease), he will be shunted immediately to the fever (isolation) area. Accompanying persons will not be allowed in the isolation area.

The fever screening form is updated regularly depending on the current international epidemiological situation as updated by the MOH through circulars issued to all hospitals and medical alerts to all medical practitioners in the state.

## The ED’s fever (isolation) areas

**Fig. 4:**
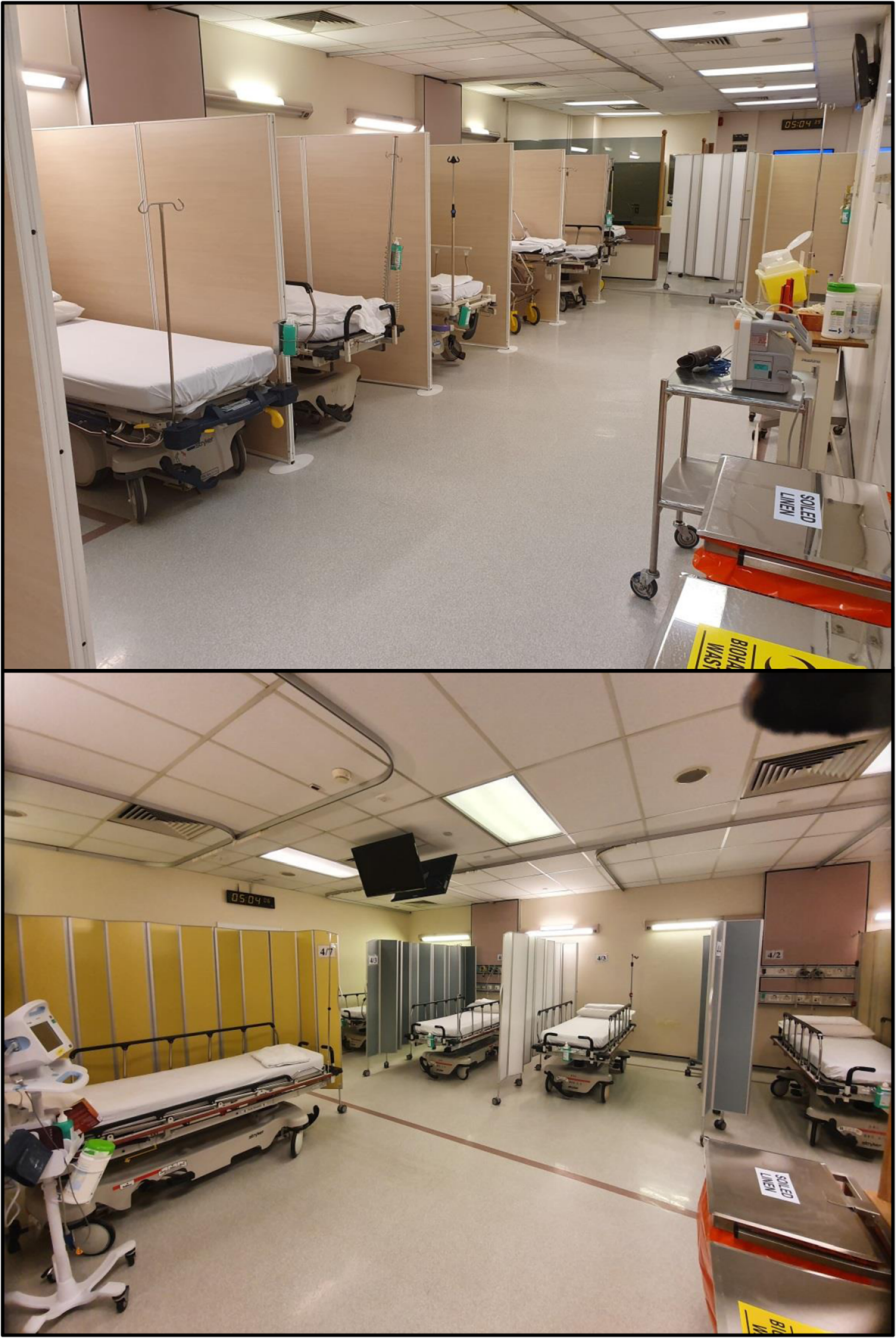
Ambulatory surgery centre as an expansion of ED fever area

In 2003, Singapore was one of 21 countries around the world affected by the SARS crisis [10]. Because SARS was thought to be an extremely infectious illness, an isolation area was carved out of the ambulatory section of the Department on the day the SARS outbreak was declared in Singapore. This area was made a negative pressure area with its own ventilation and airflow system. Fresh air was introduced into this area, unlike conventional air-conditioned systems which use re-circulated indoor air for an efficient cooling process. All effluent air from each of the rooms in the fever section would be diverted to the outer atmosphere through ducts in the roof equipped with HEPA (high-efficiency particulate air) filters and after they had been ultraviolet radiated. The section has 11 beds, its own triage room, X-ray suite, toilets, registration counter and protected access to the Department’s own pharmacy. This section had been used frequently during the SARS outbreak and during subsequent international outbreak situations such as the H1N1 (2009), MERS CoV (from 2012 onwards) and the Ebola crisis (from 2014 onwards). In addition, four of the resuscitation bays in the ED’s resuscitation zone had been also equipped with negative pressure facilities. By early February 2020, owing to increasing numbers of COVID-19 cases from local transmission, the number of patients presenting at the ED fitting the high-risk criteria at fever screening quickly overwhelmed these areas. To meet this challenge, the hospital cancelled elective procedures and transferred the management of the adjacent 40-bedded Ambulatory Surgery Centre, to the ED (Fig. 4). This area was also provided with isolation facilities. Therefore, at the start of ramped-up operations, the ED had a total of 51 isolation beds.

In the first 2 weeks of the outbreak situation, the isolation areas received up to 110 patients daily. All staff working in these areas are clothed in full PPE, including N95 masks, splash-proof goggles, disposable water-resistant isolation gowns with knitted cuffs, shoe covers, gloves and head covers when attending to suspect COVID-19 patients. Isolation gowns and gloves have to be changed in between patients. Every bed there has its own alcohol rub bottles and other common user items that would be required for patient care.

## The forward/flu screening area

**Fig. 5:**
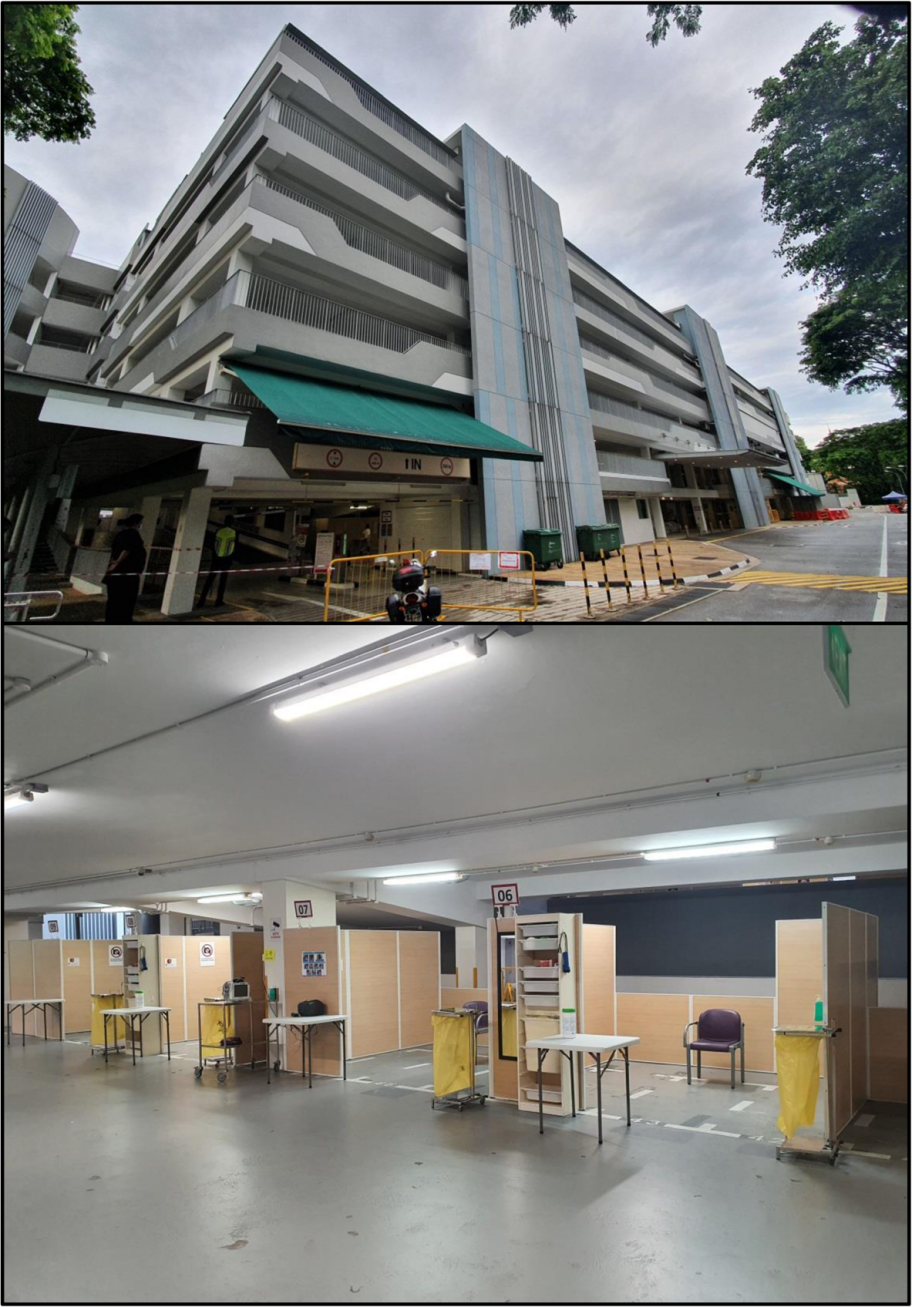
Exterior facade and interior layout of patient care area in the forward screening area

The surge in the number of fever patients being referred to the EDs for screening raised concern that the isolation areas would be overwhelmed. The hospital activated a forward fever screening area (also dubbed flu screening area) to screen ambulant patients being referred to the hospital either by primary care clinics or coming on their own accord. The need for such an area had been anticipated soon after news of the outbreak in China had broken. The hospital had prepared its multi-storey car park located in the campus, about 800 m away from the ED, to be converted to an FSA for ambulant patients. This FSA could cater to about 73 ambulatory patients at a time. For this purpose, it was fitted with improvised cubicle partitions, electrical re-wiring for computer network access, support for electrical devices, space for placement of medical equipment and supplies and facilities for command room, restrooms, pantry and rest areas (Fig. 5). The hospital had trained staff of the ED and other clinical and non-clinical departments to manage the FSA and had conducted full-scale, real-time exercises to practice the procedures there in the 2 months before the circuit breaker was declared. Once activated on 20 March 2020, the FSA managed up to about 80 patients daily.

## Management of resuscitation, critical care trolley and ambulatory areas

On a normal day, the ED is manned by three shifts of doctors and nurses over a 24-h period. Owing to the increase in the number of patients coming to the ED, the diversion of ambulances from the main hospital that was housing the majority of COVID-19 patients and the opening up of more areas being manned by the ED, shortage of ED manpower became a real concern. The hospital provided additional Residents to help in the staffing of the ED. This was made possible because elective surgical procedures in the hospital were curtailed leading to the availability of staff for needed critical areas. At the ED, staff moved to a 12-h shift system to maximize the numbers available per shift. In addition, the Department divided the staff into five separate teams, with each team assigned covering multiple areas during each 12-h shift. The longer shift hours allowed better coverage of the various areas of need. The intervals between each 12-h shift allowed each team to adequately recuperate from a long busy shift and ready to work non-stop for their next clinical session. During this period, there was a need to ensure that the ED remained operational in the event any ED staff contracted COVID-19. The team-based system ensures that if one member of a team were to turn positive for the virus, it will be more likely that only members of that team may be affected and would need to be taken out of action. The other teams would be likely to remain clean. This would minimize threats to the integrity of ED staffing. Vacation leave was also cancelled for all grades of staff across the hierarchy of the hospital.

When performing aerosol-generating procedures for any patient, the minimal requirement was an N95 mask with goggles or powered air-purifying respirator (PAPR). All hospital staff had undergone prior N95 mask fitting in the weeks following the first indication of the outbreak in China. Those who needed to use the PAPR had to attend a refresher recertification course on its use.

The non-fever areas of the ED are split into 3 separate areas—the resuscitation room for severely ill patients (priority 1), the critical care trolley area for moderately ill patients (priority 2) and the ambulatory area for patients with mild conditions (priority 3).

All patients brought to the six-bedded resuscitation room were regarded as potentially infected with COVID-19 because of likely difficulty in obtaining a clear contact history from them. When the history suggested possible contact, the patient would usually be managed in one of the four resuscitation bays that were isolation-type. X-rays for these patients were done in the bays which were fitted with ceiling-mounted X-ray tubes. The exterior of the equipment was cleaned soon after each use so that the X-ray tubes would be available for the next patient. The radiology department also reported on the X-rays within 1 h of the images being taken. Blood, throat and nasal samples were all handled with strict infection-control precautions by the staff. In the event of a large number of severely ill patients, specified bays in the priority 2 area were used for resuscitation. Resuscitation equipment and monitors were made available in these areas.

Provision was made for the occasional patient scheduled for discharge from the ED but with a need to get a naso-oropharyngeal swab done. All such patients were also provided with 5 days of medical leave and a stay-home order for that period. This order was backed up legally by the state.

To minimize the risk of ED staff cross-contaminating the community after work, personal scrubs were no longer allowed. Hospital-issued scrubs were organised by the hospital’s linen department twice daily in large trolleys. After their clinical shift, all staff would proceed to the restrooms for a shower, leave their used hospital-issued scrubs in allocated bins, change into their civilian clothes and straight out to the car park or the public transportation systems without moving into the clinical areas again. After use, the scrubs are sent to the linen department for disinfection and washing.

## The role of the emergency observation ward

In normal times, the 40-bedded emergency observation ward would be managing patients on protocolized care of between 8 to 23 h. Some would be on shorter observation stay while waiting for investigations results. Others may be waiting for an inpatient bed. When DORSCON orange was declared, there was concern with distancing requirements and a need to keep available beds in the observation ward for patients awaiting a bed and especially for those requiring short-term observation. Therefore, prolonged observation protocols were suspended and arrangements made with multiple clinical departments to conduct reviews of patients undergoing shorter sub-4-h observation, at their specialist outpatient clinics. This released quite a number of observation beds.

With increasing involvement of dormitory workers in the infection, there was an expectation that a large number of such persons may be brought to the ED and requiring inpatient admission. This would require a larger number of isolation beds and even resuscitation beds within the ED. For this contingency, approximately half the observation beds have been also assigned for priority 2 use and the logistics requirements also put in place. This flexibility in use of the emergency observation ward has been utilized in previous mass casualty situations such as during a chlorine gas accident and a mass organophosphate poisoning incident.

## Disposition of patients

At the beginning of the outbreak, there were differences in the risk stratification methods used by Emergency Physicians and Infectious Diseases Specialists. This could potentially affect patient disposition in the hospital. To mitigate this issue, the ED teamed up with the ID department and drew up disposition protocols for possible infectious disease patients. These protocols were based on the latest national epidemiological risk and patient assessments and served to standardize management of possible infectious disease patients based on their probability of having COVID-19. They also better ensure that all doctors follow the case definitions for the disease, including the criteria for diagnosis.

The principles of disposition are as follows:

1. The ED doctor decides if COVID-19 swabbing is required based on the latest disposition protocols.
2. Patients who are well enough for discharge are given stay-home and self-quarantine orders, which are enforced until swab results are made available.
3. If hospital admission is necessary, patients are risk stratified (low, medium or high) based on their history, radiological and blood investigations.
4. High-risk patients are isolated in single-bedded rooms.
5. Medium-risk patients are placed in well-ventilated common wards, with beds more than 3 m apart and separated with partitions.
6. Low-risk patients are placed in well-ventilated wards with beds, at least 2 m apart.

The risk stratification process is regularly updated and refined as more information about the disease becomes available. The updated protocols are put up daily in all the care areas of the Department for doctors to refer to when in doubt.

## Emergency department organisation and leadership during the outbreak

**Fig. 6:**
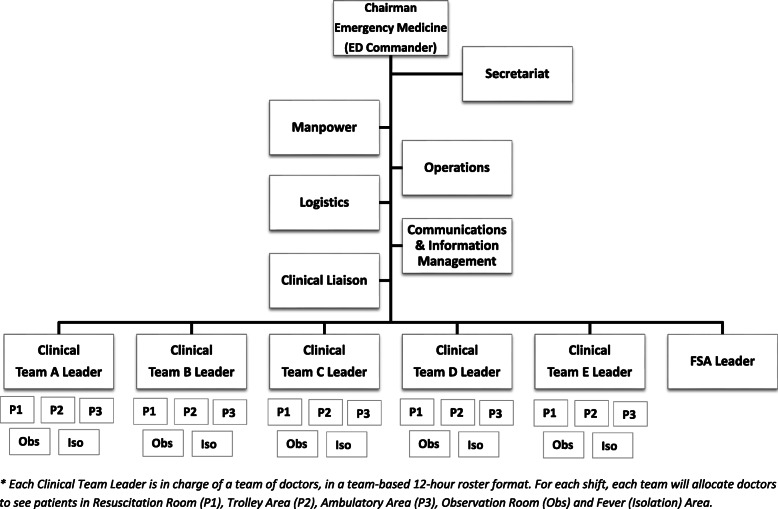
SGH emergency department leadership and operational organisation for COVID-19 outbreak. Each clinical team leader is in charge of a team of doctors, in a team-based 12-h roster format. For each shift, each team will allocate doctors to see patients in resuscitation room (P1), trolley area (P2), ambulatory area (P3), observation room (Obs) and fever (isolation) area

Ensuring that patients coming to the ED undergo a relatively seamless and hassle-free process requires clear organisation and leadership. This is especially important during an unpredictable crisis situation, like the COVID-19 pandemic. For SGH ED, leadership was re-organised at the beginning of operations to allow for this (Fig. 6). The need to split the ED into multiple teams and serve multiple areas required a good command and control system. Operational command and control was established with the Department Chair as the ED Commander, directly commanding the five clinical operational teams and the FSA team. Each of these teams was led by a Senior Emergency Physician. Within each operational team, members were assigned to the various patient care areas within the Department. These were the fighting troops meeting the patients presenting to the Department head-on and managing them within the framework of the operational guidelines and protocols provided by the Department.

Supporting the ED Commander was a staff team covering five areas, each team led by other senior Emergency Physicians. Assistance was also provided by an executive secretariat led by the Department’s administrative manager. The five areas covered were as follows:

- Manpower
- Operations
- Logistics
- Communications and information management
- Clinical liaison

### Secretariat

The administrative manager coordinated the work of the secretaries, executive and administrative assistants to ensure that all operational charts and workflow information were updated and logistics arrangements executed. The secretariat set up the areas for key meetings held in the ED with precautions taken for social distancing and use of appropriate PPE during such meetings. Designated areas were set up for meals provided to the staff. Sourcing for PPE and appropriate disposal arrangements were made by them.

### Manpower

As mentioned earlier, separate operational teams were set up from the manpower available within the Department. Critical manpower shortfalls were identified early and liaison established so that requirements for medical, nursing and patient care assistant staff were addressed from within the hospital without having to be sourced from the general community. The manpower team ensures that rostering arrangements are worked out and all staff promptly informed of any changes. Their requirements for the various forms of PPE were determined, and this information passed on to the secretariat. Staff with special needs had arrangements specially arranged for them by the team. The manpower team also addressed the often-forgotten area of staff welfare and wellness. A peer counselling team was set up within the Department to address issues pertaining to stress management during the conduct of departmental operations. Peer support leaders have also been appointed in every clinical team to facilitate the conduct of debrief sessions at individual and team level, whenever necessary.

### Operations

The constantly evolving COVID-19 situation, clinical and operational protocols and arrangements required frequent review and amendments. The operations team would work out the needed changes and discuss with relevant members of the ED via telephone, email, WhatsApp™, TigerConnect™ or ZOOM™ videoconferencing. After final discussion with the ED Chair, the operations team will produce instructions and revised protocol charts to be disseminated to the teams. In addition, the operations team would monitor the clinical load at each of the areas being covered by the ED. Feedback was regularly received during the various shifts of work to adjust operational plans and address needs. The operations team would also work closely with the logistics team to ensure that resources needed by the clinical teams were requisitioned early and sent to the required areas in timely fashion.

### Logistics

The need for logistical items special to disease outbreak situations is often not well appreciated in healthcare institutions until the eleventh hour. Previous experience with the SARS outbreak 17 years ago, H1N1, MERS and Ebola situations helped in the logistics planning this time around. Immense preparations were made for those events, and the team was well-aware of the need to anticipate requirements and plan for these early. Once the situation is evolving, logistical arrangements may not always be executed smoothly. The team, therefore, listened to feedback from the clinical teams and ensured that any hitches in supplies were rectified promptly. Logistical needs were not only in the areas of medical equipment and pharmaceuticals, but also for internal logistics, such as appropriate PPE, meals and even special meals for staff. These have helped to keep morale high during the disease outbreak period.

### Communications and information management

During any disaster, communication is extremely important to ensure that units are apprised as to what is happening, able to effectively transmit relevant messages and advise those in need. Lack of communication or misinformation can have dire consequences on patient care, staff safety and morale. The ED has been using multiple modes of communication to enable the departmental leadership to communicate with the staff. These include email, creation of WhatsApp™ groups, TigerConnect™, Workplace by Facebook™, daily routine instructions and departmental bulletins, in addition to face-to-face briefings albeit with PPE. Every shift begins with a short clinical team meeting, after which information is shared with the team members. Owing to the tremendous amount of information that needs to be exchanged and to ensure that staff stay updated on the latest information and able to easily communicate with all levels of the Department, the ED has applied the discipline of ensuring better security of patient information during this outbreak period. All staff have been advised that no clinical or treatment information on patients can be shared via WhatsApp and that only secure applications are to be utilized for such.

Of course, the ED Commander also attends daily meetings with the hospital’s Emergency Preparedness Task Force and represents the ED in all dealings with senior management and with the MOH, whenever, necessary.

### Clinical liaison

Liaising with the other clinical departments in the hospital by the ED leadership has become the norm for many years in the hospital. During the COVID-19 outbreak, such liaison has been very helpful in drawing up departmental infectious disease management protocols, patient disposition protocols, arrangements for subsequent early follow-up of patients being discharged from the ED, and in adjustments of current arrangements the department has with the other 36 clinical departments in the hospital for various aspects of patient care. These have helped to better ensure that the ED’s management of suspect and confirmed COVID-19 patients stays in line with the hospital’s and with the state’s overall management framework. On-line clinical decision rules based on these algorithms have also been developed, based on the clinical algorithms created, via Microsoft© Forms accessible with QR codes. These have helped to decrease the cognitive workload and increased confidence of doctors in management of the variety of potentially infected patients presenting to the ED during this period.

## Contribution to the community during the outbreak

In addition to all of the above, and in response to calls from the hospital and from the MOH, the ED has taken the lead and mobilized a small team to go in full PPE into some of the foreign worker dormitories in the country to screen persons staying there, conduct nasopharyngeal swabs and speed up the process of early identification of COVID-19 cases, whether symptomatic or otherwise. The team has screened a few thousand such persons. This has contributed to speedier identification, isolation, safe distancing and treatment of symptomatic cases.

## Conclusion

The situation continues to evolve. With the large numbers of undifferentiated patients managed by the ED, we have identified, as of 8 May 2020, 820 patients with the COVID-19 infection [9]. Since the start of the outbreak, not a single member of the staff of the SGH ED has come down with the illness during this period. The various measures undertaken by the staff have helped to ensure good staff morale and strict adherence to safety procedures. We share the lessons we have learnt during the outbreak with others who manage EDs around the world and look forward to seeing the establishment of an international sharing and communication network with EDs worldwide. We all need to learn from each other.

## Data Availability

Data sharing is not applicable to this article as no datasets were generated or analysed during the current study. All data quoted in this paper are in the public domain.

## Abbreviations

COVID-19: Coronavirus disease 2019
WHO: World Health Organization
SGH: Singapore General Hospital
ED: Emergency department
DORSCON: Disease Outbreak Response System Condition
PPE: Personal protective equipment
FSA: Forward screening area
